# Synthesis of Methane Hydrate from Ice Powder Accelerated by Doping Ethanol into Methane Gas

**DOI:** 10.1038/s41598-019-48832-8

**Published:** 2019-08-26

**Authors:** Yen-An Chen, Liang-Kai Chu, Che-Kang Chu, Ryo Ohmura, Li-Jen Chen

**Affiliations:** 1 Department of Chemical Engineering, National Taiwan University, Taipei, 10617 Taiwan; 2 Department of Mechanical Engineering, Keio University, 3-14-1 Hiyoshi, Kohoku-ku, Yokohama 223-8522 Japan

**Keywords:** Natural gas, Chemical engineering

## Abstract

Clathrate hydrate is considered to be a potential medium for gas storage and transportation. Slow kinetics of hydrate formation is a hindrance to the commercialized process development of such applications. The kinetics of methane hydrate formation from the reaction of ice powder and methane gas doped with/without saturated ethanol vapor at constant pressure of 16.55 ± 0.20 MPa and constant temperature ranging from −15 to −1.0 °C were investigated. The methane hydrate formation can be dramatically accelerated by simply doping ethanol into methane gas with ultralow ethanol concentration (<94 ppm by mole fraction) in the gas phase. For ethanol-doped system 80.1% of ice powder were converted into methane hydrate after a reaction time of 4 h, while only 26.6% of ice powder was converted into methane hydrate after a reaction time of 24 h when pure methane gas was used. Furthermore, this trace amount of ethanol could also substantially suppress the self-preservation effect to enhance the dissociation rate of methane hydrate (operated at 1 atm and temperatures below the ice melting point). In other words, a trace amount of ethanol doped in methane gas can act as a kinetic promoter for both the methane hydrate formation and dissociation.

## Introduction

Clathrate hydrates are ice-like nonstoichiometric crystalline compounds formed by guest molecules encapsulated in the hydrogen bonded water cages at elevated pressures and low temperatures^1^. There are three main distinct crystalline forms of structure I (sI), structure II (sII), and structure H (sH) depending on the size and composition of guest molecules. Methane or carbon dioxide can form sI hydrates, which consist of two small cages (5^12^) and six large cages (5^12^6^2^) in the unit cell formed by 46 water molecules and can give a maximum of 174.6 m^3^ (STP) of gas in 1 m^3^ of clathrate hydrates. Nitrogen or oxygen can form sII hydrates, which consist of sixteen small cages (5^12^) and eight large cages (5^12^6^4^) in the unit cell formed by 136 water molecules and can give a maximum of 174.9 m^3^ (STP) of gas in 1 m^3^ of clathrate hydrates^1,2^. 

Clathrate hydrates have been widely explored and investigated since 1930s due to the flow assurance and safety concerns in oil and gas pipelines^3^. Alcohols, electrolytes, polyols, and ethylene glycols have been well studied and verified as thermodynamic inhibitors in oil and gas industry to prevent gas hydrate formation from plugging oil and gas pipelines and production wells^4–10^. The addition of a thermodynamic hydrate inhibitor would shift the hydrate stability zone to lower temperature and higher pressure region to ensure no hydrate formation in the pipelines.

In addition to the thermodynamic phase behavior, kinetic studies of clathrate hydrate formation have attracted many attentions. Note that the rate of clathrate hydrate formation and dissociation are important and necessary information for the practical applications, such as desalination, gas separation, gas storage and transportation^11–17^. It is well understood that alcohols and ethylene glycols are widely used as thermodynamic inhibitors in offshore hydrate control operations. However, alcohols have also been verified as kinetic promoters of clathrate hydrate formation from aqueous solutions^18,19^ or ice particles^20–23^.

Yousif^18^ pointed out that methanol and ethylene glycol tend to enhance the rate and amount of hydrate formation when present in small concentrations. For example, ethylene glycol with a concentration of as low as 2 wt% shows a dramatic increase in the gas consumption, i.e., a dramatic increase in hydrate formation. Abay and Svartaas^19^ measured the induction time of methane hydrate with ultralow concentration (ranging from 1.5 to 20 ppm by weight) methanol aqueous solution and determined the rate of nucleation and induction time by fitting the parameter of the nucleation probability distribution function. Their results indicated that methanol can act both as an inhibitor and as a promoter, depending on the methanol concentration, for the structure I methane hydrate formation from liquid water.

Bobev and Tait^21^ performed *in situ* time-of-flight neutron powder diffraction experiments to investigate the formation rate of the CO_2_ and CH_4_ hydrate from frozen deuterated water + methanol mixture (up to 20 vol%) and found out that methanol could act as a kinetic promoter for the sI hydrate. McLaurin *et al*.^20^ synthesized methane hydrate from powder of frozen water + methanol (0.6–10 wt%) or + ammonia (0.3–2.7 wt%) mixtures at 253 K and 124 bar. By monitoring the pressure drop in 22 h, they pointed out that both methanol and ammonia could enhance the formation rate of methane hydrate, and the optimum concentration were 1.2 wt% and 1.4 wt% for methanol and ammonia, respectively. Besides, the hydrates formed in this manner were characterized to be structure I by PXRD and Raman spectrum. Amtawong *et al*.^22^ injected propane gas into a pressure cell to react with methanol-doped ice particles to form propane hydrates. By comparing the pressure variation profile between empty cell and cell filled with methanol-doped ice particles, the propane uptake rate could be quantified as the hydrate formation rate. Their results also indicated that with small quantities of methanol the propane hydrate formation rate can be substantially enhanced.

It should be pointed out that all the gas-solid reactions for hydrate formation accelerated by methanol discussed above methanol was introduced into the system as a solid phase, powdered frozen water + methanol mixture^20–22^. In this study, ethanol was used to enhance the rate of hydrate formation from the reaction of pure ice powder with “ethanol-doped methane gas”. First of all, around 2 ml liquid ethanol was added at the bottom of reaction tank. There was no direct contact between liquid ethanol and pure ice powder. Ethanol would evaporate into vapor phase to mix with methane gas to form the “ethanol-doped methane gas”. Those ethanol molecules in the vapor phase may then physically adsorb onto the surface of ice powder. It is interesting to find out that such a trace amount of ethanol could substantially enhance the methane hydrate formation rate. Furthermore, the reaction temperature effect on the methane hydrate formation was also examined over the temperature ranging from −14.0 to −1.0 °C which cover the temperature range of most effective self-preservation phenomena observed by Stern *et al*.^23,24^ Finally, the effect of this trace amount of ethanol on the dissociation rate of methane hydrate in the dissociation process operated at 1 atm and temperatures below the ice melting point was also examined and discussed.

## Results and Discussion

**Figure 1:**
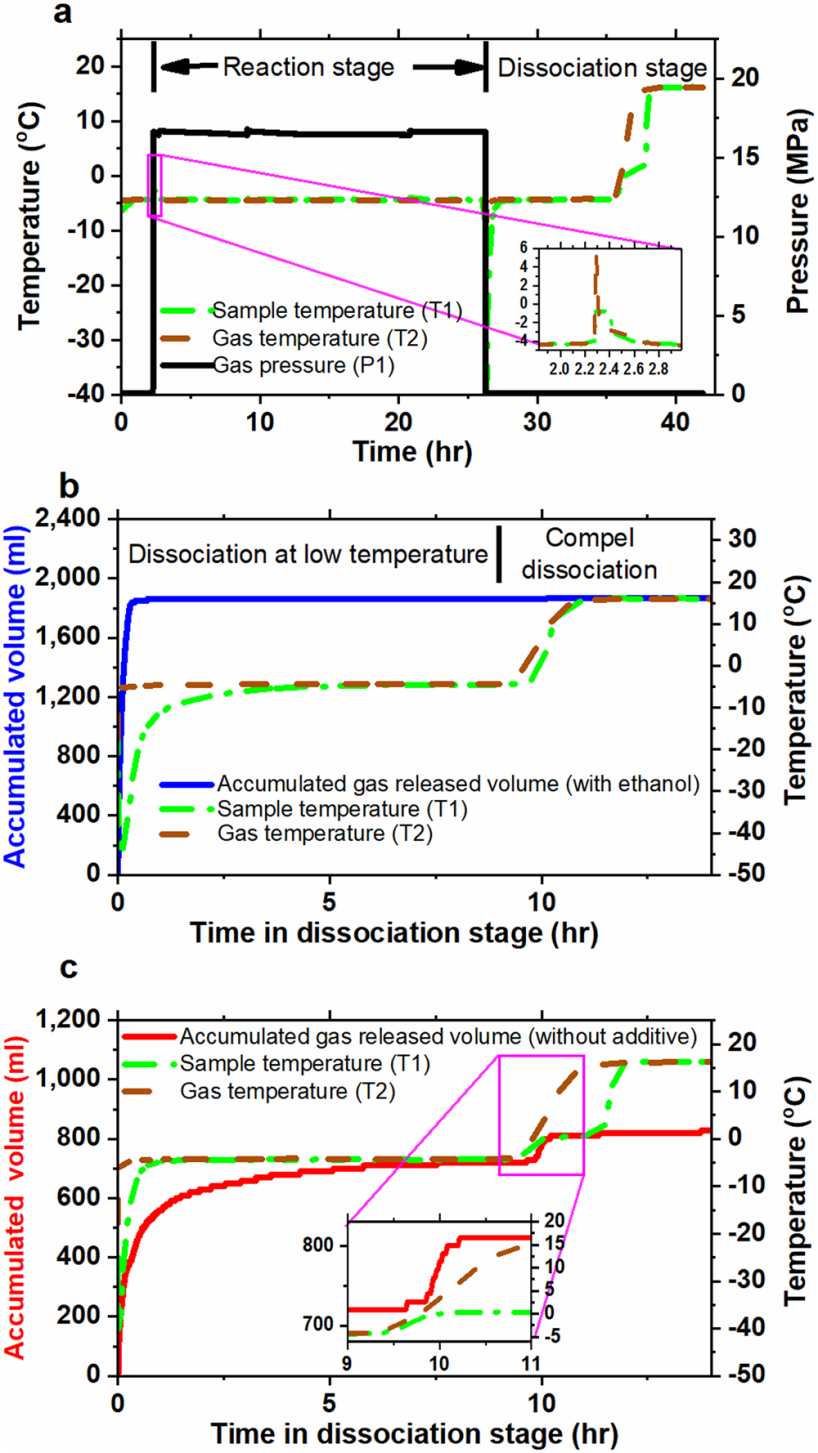
Variation of temperature, pressure, and flow rate as a function time. (**a**) Variation of pressure, gas temperature and sample temperature in the reaction vessel as a function time for the system without ethanol. (**b**) Accumulated volume of gas released from hydrate dissociation in the system with 2 ml ethanol added, but not direct contact with ice powder, as a function of time in the dissociation stage. (**c**) Accumulated volume of gas released from hydrate dissociation in the system without additive (ethanol) as a function of time in the dissociation stage. Green dashed-dotted line, the sample temperature; brown dashed line, the gas temperature in the reaction vessel; and black solid line, the pressure in the reaction vessel; red line and blue line, accumulated volume of gas released from hydrate dissociation in system, respectively, without and with ethanol added.

The variation of sample temperature, gas temperature and pressure as a function of time throughout the experiment for a prescribed reaction temperature of −4.3 °C is demonstrated in Fig. 1(a). The experimental operation process can be divided into three stages.

1. Preparation stage (0 ~2.3 h in Fig. 1(a))
  After placing ice powder sample container into the reaction vessel and sealing the reaction vessel, the temperature of the reaction vessel was controlled at the prescribed temperature (of −4.3 °C in this case) by the ethylene glycol aqueous solution bath. Preparation stage lasted until the temperature difference between sample and gas temperatures was less than 0.3 °C.
2. Reaction stage (2.3~26.3 h in Fig. 1(a))
  While the reaction vessel was pressurized by methane from the reservoir vessel, the temperature rose due to the work of high pressure methane done on the system in the reaction vessel. Besides, gas hydrate formation is an exothermic process, therefore the sample temperature would increase further (see the inset in Fig. 1(a)). Usually, it took a certain time for the sample temperature to go back to its prescribed reaction temperature, due to the exothermic effect and low thermal conductivity. However, the sample temperature or gas temperature in the reaction vessel would not excess the equilibrium temperature of methane hydrate, which is 17.46 ± 0.1 °C (calculated from a predictive thermodynamic model composed of COSMO-SAC model and the modified van der Waals and Platteeuw model^25,26^). The pressure would slightly decrease due to the consumption of methane gas for hydrate formation. However, the pressure always maintained at 16.55 ± 0.20 MPa by pneumatic valve. (see the method section)
3. Dissociation stage (26.3~42 h in Fig. 1(a))
  The dissociation process was operated at atmospheric pressure and the system temperature was kept constant at the reaction temperature used to convert ice into hydrate for 9 h and then the system temperature was increased up to 15 °C to ensure dissociating all the hydrate and ice remaining inside the system. The dissociation stage was kicked off by depressurizing the reaction vessel to atmospheric pressure. As expected, the temperature dropped in the depressurizing process owing to the work done by the system to release high pressure methane to the atmosphere. The sample temperature would decrease further because the hydrate dissociation process is an endothermic process. It also took a certain time for the sample temperature to relax back to its prescribed reaction temperature. When the system in the reaction vessel was depressurized down to atmospheric pressure (usually took less than 1 minute), the wet gas flow meter then took over to monitor the amount of gas released from the dissociation of methane hydrates as a function of time, as illustrated in Fig. 1(b,c). When ethanol had been added into the reaction vessel, the gas released rapidly in the first hour and did not release any gas during the system being heated up to 15 °C, as the blue solid line illustrated in Fig. 1(b). However, for the experiments without ethanol added, methane hydrate only partially dissociated at the reaction temperature and substantial amount of methane hydrate was forced to dissociate after the system being heated to 15 °C, as the red solid line illustrated in Fig. 1(c). It is obvious that the time lag of the sample temperature compared to the gas temperature in the system without ethanol (see Fig. 1(c)) is more dramatic than that in the system with ethanol added (see Fig. 1(b)) due to the dissociation of methane hydrate and remaining ice.

### Methane hydrate conversion ratio and dissociation in the first hour

The total amount of released gas measured by the wet gas flow meter can be applied to determine the total amount of methane hydrate formation under the assumption of the hydration number of 5.75, full occupancy of methane in large and small cages of structure I hydrate. Later on, we will discuss all the methane hydrates formed by using the ice seed method with/without ethanol were structure I hydrate. Furthermore, the hydrate conversion ratio *C*_*r*_ is defined as the mass fraction of ice being transformed into methane hydrate and calculated by the following equation,

1 $${C}_{r}=\frac{P{V}_{total}}{RT}/\frac{{m}_{i}}{{n}_{H}{M}_{w}}$$

where *P* is atmospheric pressure (=101325 Pa), *V*_*total*_ is the total volume of released gas measured by the wet gas flow meter (m^3^), *R* is the gas constant (=8.314 J/mol K), *T* is the temperature of gas measured in the wet gas flow meter (K), *m*_*i*_ is the initial mass of ice loaded into the ice powder sample container (g), *n*_*H*_ is the hydration number, assumed to be 5.75 in this study, and *M*_*w*_ is the molecular weight of water (=18.02 g/mol).

**Figure 2:**
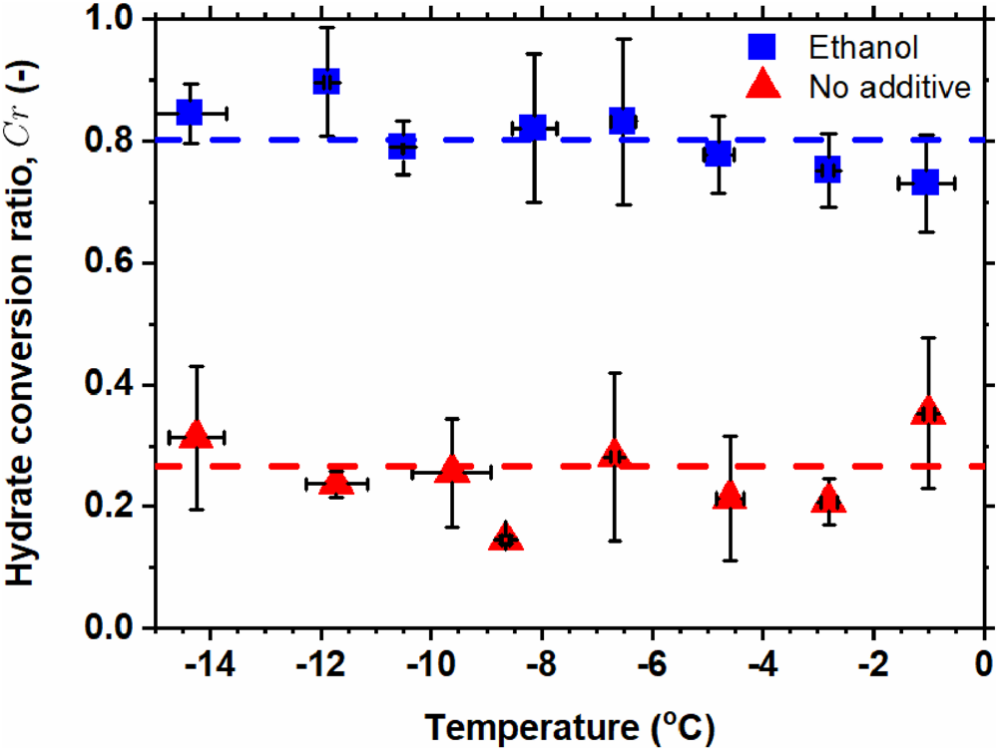
Conversion ratio of methane hydrate from ice for reaction time of 24 h at different temperatures. Red triangle, the conversion ratio for the system without ethanol added; and blue square, the conversion ratio for the system with ethanol added. Blue and red dashed lines stand for the average values of the conversion ratio for the system, respectively, with and without ethanol added.

The conversion ratios of ice/CH_4_ hydrate with and without ethanol additive for reaction time of 24 h at different temperatures are shown in Fig. 2. It is interesting to find out that the conversion ratio is independent of temperature ranging from – 14 to – 1 °C. The average values of the conversion ratios of ice/CH_4_ hydrate with and without ethanol additive are 0.801 and 0.266, respectively, as the blue and red dashed lines illustrated in Fig. 2. The conversion ratios of the systems with ethanol were consistently much higher than that without ethanol. That implies the ethanol additive can act as a kinetic promoter for hydrate formation.

**Figure 3:**
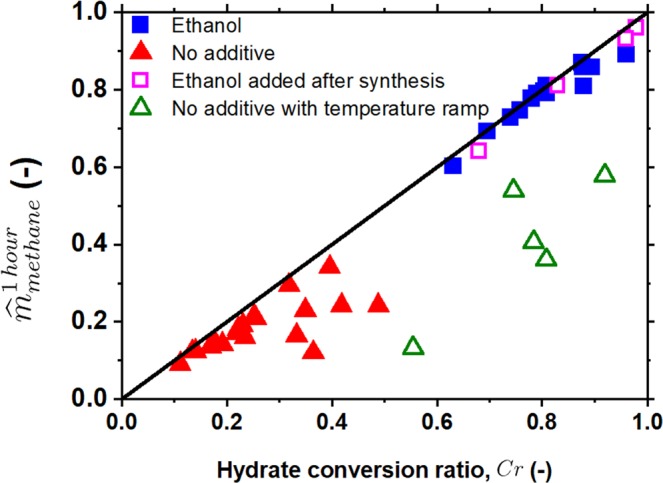
The relation between the mass fraction of gas released in the first hour of the dissociation process and the hydrate conversion ratio. Red filled triangles, the results without ethanol added in the system; blue filled squares, the results with ethanol added in the system; green open triangles, the results without ethanol added but with temperature ramping process to enhance the conversion ratio; violet open squares, the results with temperature ramping process and ethanol injected at one hour before dissociation process.

Figure 1(b) shows that the most of methane hydrate dissociated in the first hour for the system with ethanol, but it is not the case for the system without ethanol as the red line illustrated in Fig. 1(c). To examine the effect of ethanol on the dissociation process, the volume of gas dissociated in the first hour of the dissociation process (*V*_*1 hour*_) was normalized by the total volume of gas released from the completely converted hydrate, that is defined as the mass fraction of gas released in the first hour ${\hat{m}}_{methane}^{1\,hour}$. The reference “completely converted hydrate” is defined as the condition that all the ice powder was completely converted into methane hydrate, that is the same reference used in Eq. (1). The mass fraction of gas released in the first hour ${\hat{m}}_{methane}^{1\,hour}$ could be calculated by

2 $${\hat{m}}_{methane}^{1\,hour}=\frac{P{V}_{1hour}}{RT}/\frac{{m}_{i}}{{n}_{H}{M}_{w}}$$

where *V*_*1 hour*_ is the volume of gas released in the first hour of the dissociation process. The variation of the ${\hat{m}}_{methane}^{1\,hour}$ as a function of the hydrate conversion ratio is illustrated in Fig. 3. The experimental results of ${\hat{m}}_{methane}^{1\,hour}$ for the systems with ethanol added were always very close to the diagonal line as illustrated in Fig. 3, which means the methane hydrate almost completely dissociated in the first hour of the dissociation process, no matter how high the conversion ratio was. While the accumulated amount of gas released in the first hour for the systems without ethanol were much lower than the total amount of gas storage in hydrate whenever the conversion ratio was higher than 0.30, as the red filled triangles deviated from the diagonal line illustrated in Fig. 3. The slower dissociation rate might attribute to the self-preservation behavior. It seems that the outermost shell of ice particles had been transformed into a thin layer of methane hydrates for the systems without ethanol. This thin layer of methane hydrate of the hydrate conversion ratio less than 0.30 would almost fully dissociated in the first hour of the dissociation process for the systems even without ethanol.

In addition, the constant reaction temperature process in the reaction stage is replaced by the temperature ramping process to enhance the methane hydrate conversion ratio for the system without ethanol added^27^. The temperature ramping process was designed as the system temperature was maintained at −2.3 °C for 24 h, then slowly raised up to 13.6 °C in 6 h, maintained at 13.6 °C for 8 h and then slowly decreased down to −2.3 °C in 6 h. Indeed, the methane hydrate conversion ratio was dramatically enhanced even up to 0.90 by using the temperature ramping process, as those green open triangles illustrated in Fig. 3. Similar to the systems with the hydrate conversion ratio higher than 0.30 via the constant reaction temperature process, the methane hydrate only partially dissociated in the first hour of the dissociation process as those green open triangles deviated from the diagonal line illustrated in Fig. 3.

Furthermore, the temperature ramping process was applied to enhance the methane hydrate formation in the absence of ethanol (at least C_r_ = 0.7) and then ethanol (around 2 ml) was injected into the bottom of the reaction vessel (also no direct contact between ethanol liquid and methane hydrates) by using a high pressure syringe pump at 1 hour before starting depressurization stage. It is interesting to find out that methane hydrates almost dissociated completely in the first hour of dissociation stage with the introduction of ethanol after the reaction stage, as violet open squares shown in Fig. 3. Note that ethanol still can act as a “kinetic promoter” to enhance the dissociation rate of methane hydrates even ethanol is not introduced beforehand in the process of methane hydrate formation. That makes ethanol a perfect kinetic promoter for methane hydrate dissociation.

**Figure 4:**
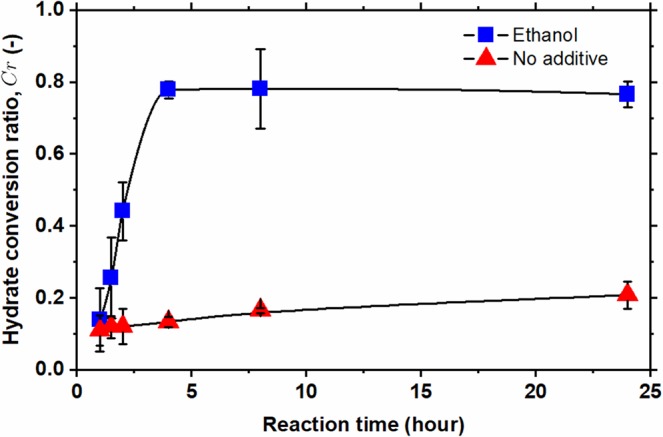
The conversion ratio of methane hydrate from ice under the condition of a fixed reaction temperature of −2.7 °C at different reaction (formation) times. Blue squares and red triangles stand for the hydrate conversion ratio for the systems, respectively, with and without ethanol added. Solid lines are guides of eyes.

All the data illustrated in Fig. 3 demonstrate that ethanol can dramatically enhance the dissociation rate of methane hydrates. What about can ethanol enhance the reaction rate of methane hydrate formation? We carried out the experiments of methane hydrate formation with/without ethanol at various reaction times, instead of reaction time of 24 h, at a fixed reaction temperature – 2.7 °C, that enables us to observe the kinetic effect of ethanol on the methane hydrate formation (or conversion). Figure 4 illustrates the variation of the hydrate conversion ratio for the systems with (blue squares) and without (red triangles) ethanol as a function of reaction time. The conversion ratio of methane hydrate without ethanol were about 0.11 for one-hour reaction time and then rose gradually to 0.20 for 24 h reaction time. For the system with ethanol added, the conversion ratio for reaction (formation) time of one hour was 0.16 and raised rapidly along with an increase in reaction time from 1 to 4 h and became stable around 0.80 for reaction time longer than 4 h. It is rather obvious that ethanol does enhance the reaction kinetics of methane hydrate formation.

**Figure 5:**
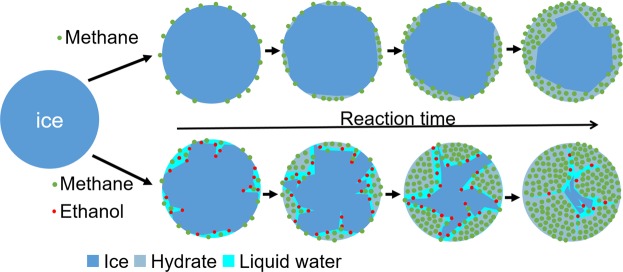
Schematic of the reaction process of methane hydrate conversion from ice in the absence (upper row) and presence (lower row) of ethanol.

Despite of the fact that liquid ethanol did not directly contact with ice powder, vapor pressure of ethanol at temperature ranging from – 15 to 0 °C is around from 499.3 to 1568.1 Pa (calculated from the Antoine equation of Dortmund Data Bank). For the system pressure of 16.65 MPa methane, the ethanol concentration (in terms of mole fraction) in the vapor phase is always lower than 0.000094 (or 94 PPM). It has been pointed out that the quasi-liquid layer (QLL) on the surface of ice powder plays an important role in the formation of clathrate hydrate^28–30^. It is plausible to conjecture that a small amount of ethanol in the vapor phase would adsorb on the surface of ice powder due to the molecular structure of ethanol rather similar to that of a short chain surfactant molecule. In addition, it is well understood that addition of ethanol into water would dramatically suppress the melting temperature of ice (water), for example, the eutectic temperature of binary ethanol + water system is as low as −124.5 °C at 1 atm (and −68.0 °C at 7800 bar)^31^. It is very likely that those adsorbed ethanol molecules would trigger nearby ice melting due to capability of ethanol for suppressing melting temperature of ice. That is, the surface of ice powder with a certain amount of adsorbed ethanol molecules would start to melt into liquid water and therefore enhance the thickness of QLL. Then the methane in the vapor phase would easily dissolve into liquid water. Not to mention the diffusivity of gas in liquid water is generally much higher than that in solid ice (For example, the diffusivity of methane in water is 1.12 × 10^−9^ m^2^/s at 283.15 K^32^, and that in ice is 8.34 × 10^−11^ m^2^/s at 270 K^33^, and that in hydrate is about 7 × 10^−15^ m^2^/s at 250 K^34^). Furthermore, the solubility of methane in ethanol aqueous solution is also higher than that in pure water and the solubility of methane increases along with the ethanol concentration^35^. Thus, these physical properties strongly imply the enhancement of rate of methane transport into liquid water and even toward the inner part of each ice particle, as schematically illustrated in Fig. 5. As a consequence, the rate of methane hydrate formation could be substantially enhanced.

For the hydrate dissociation process, after outermost layer of gas hydrate dissociated into liquid water on the surface of hydrate particles, the liquid water would freeze (since the system temperature maintained below 0 °C) to form a shielding ice shell sealing the remaining gas hydrate inside that dramatically slows down the rate of hydrate dissociation. This phenomenon is well known as self-preservation behavior^23,36–39^, as mentioned above. For systems without ethanol added, when the hydrate conversion ratio was higher than 0.3, the dissociation rate of methane hydrate was relatively fast in the very beginning of the dissociation process and then dramatically slowed down as time went on in the dissociation process, as a slow increase in the accumulated volume of gas released from hydrate dissociation (red line) illustrated in Fig. 1(c). Note that there always exists another jump (substantial amount of gas released) appeared in the curve of the accumulated volume of gas released from hydrate dissociation during the system being heated up to 15 °C and in the meanwhile the sample temperature would increase along and level off around 0 °C for a certain time period due to the dissociation of remaining ice/hydrates in the system (see the inset in Fig. 1(c)). That implies the existence of the self-preservation effect. However, whenever ethanol molecules in the gas phase were adsorbed onto the ice powder surface, the outermost layer of ice particles would melt into liquid water due to ethanol suppressing the melting temperature. Thus, the self-preservation effect was suppressed in the dissociation process of methane hydrate in the presence of ethanol, consistent with the observation of Nakoryakov and Misyura^40^ for the systems in the presence of n-propanol and isopropanol.

The hydrate conversion ratio was calculated by using equation (1), as mentioned above, under the assumption of the hydration number of 5.75 for structure I hydrate with full occupancy of large and small cages. However, Anderson^41^ indicated that the hydration number of structure I methane hydrate ranging from 5.75 to approximately 8.0 might result in a stable hydrate and suggested the hydration number of methane hydrate to be 5.90 ± 0.3. Handa^42^ suggested the hydration number to be 6.00 ± 0.01. If the hydrate number 6.00, instead of 5.75, is applied to Eq. (1), the conversion ratio would increase by a factor of 1.043 (=6.00/5.75). That is, the average conversion ratio for the systems in the absence of ethanol would become 0.278 and that in the presence of ethanol would become 0.839.

Recently, it has been pointed out^8,43,44^ that the binary ethanol-methane hydrate would form structure II hydrate, instead of structure I hydrate, with large 5^12^6^4^ cages occupied by either ethanol or methane molecules and small 5^12^ cages occupied solely by methane molecules. For example, Yasuda *et al*.^43^ applied the powder XRD analysis to estimate that the small 5^12^ cage occupancy of methane was 75% and the large 5^12^6^4^ cage occupancies of methane and ethanol were 67% and 33%, respectively, for the structure II binary ethanol-methane hydrate prepared at stoichiometric concentration of ethanol (0.06 mole fraction ethanol aqueous solution). That implies the hydration number of methane for this structure II binary ethanol-methane hydrate is 7.83. Besides, Lee and Kang^44^ also reported the hydration number of structure II binary ethanol-methane hydrate to be a function of ethanol concentration and ranging from 7.73 (ethanol 5.6 mol %) to 6.97 (ethanol 1.0 mol %) using ^13^C NMR combined with van der Waals-Platteeuw model. The average conversion ratio for the systems with ethanol added, if the hydration number of methane (7.83) is applied to Eq. (1), is estimated to be 1.09, larger than 1.00 (limitation of the complete conversion of ice into hydrate).

### Using Raman spectroscopy to determine the hydrate structure

**Figure 6:**
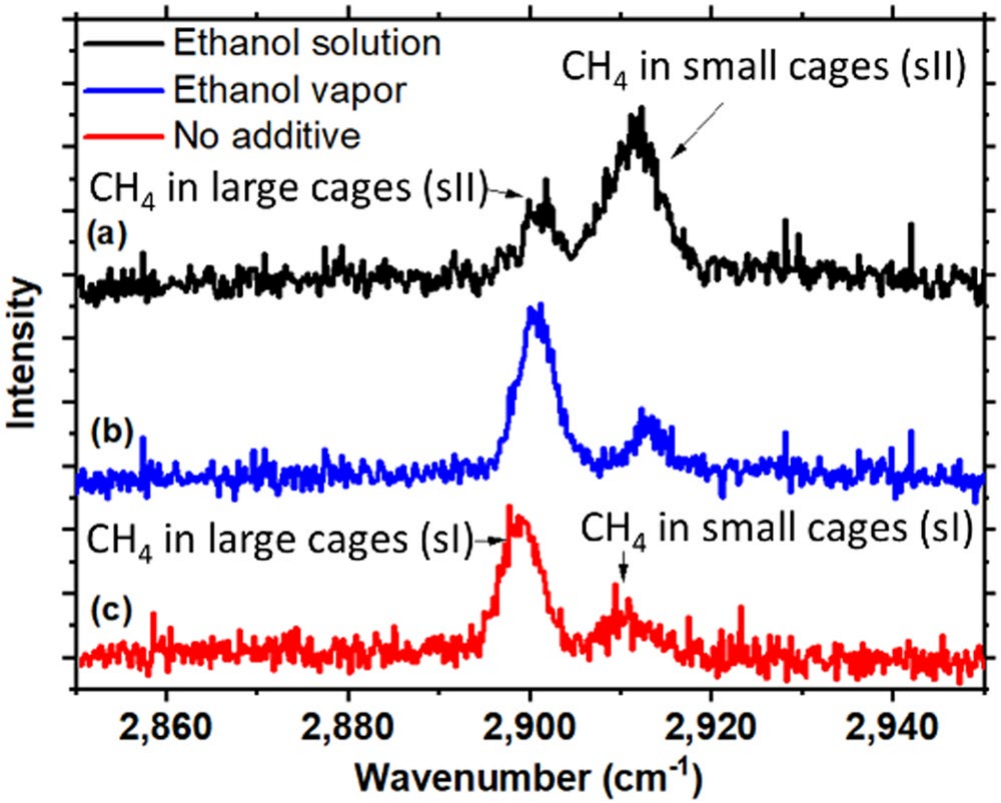
Raman spectrum of methane hydrates. (**a**) Black line indicates the binary ethanol-methane hydrate (sII) that formed by 8.6 wt% ethanol aqueous solution. (**b**) Blue line indicates methane hydrate (sI) formed by ice seed method with ethanol added but not direct contact to ice powder. (**c**) Red line indicates methane hydrate (sI) formed by ice seed method without ethanol added.

Raman spectrum experiments were conducted to determine the structure of methane hydrate formed by ice seed method in this study. The Raman shift of ν_1_ symmetric stretching of methane is about 2917 cm^−1^ and is a function of temperature and pressure^45^. The methane molecules encaged in large and small cavities of hydrates would induce different Raman shifts. The Raman shift of methane ν_1_ symmetric stretching band in small and large cavities are 2915.04 and 2904.85 cm^−1^, respectively, for sI hydrate, and 2913.73 and 2903.72 cm^−1^, respectively, for sII hydrate^46,47^. Methane hydrate sample catalyzed with ethanol vapor was synthesized via the method mentioned above. Methane hydrate sample without additive was synthesized via the temperature ramping process to enhance the hydrate conversion ratio. Figure 6 shows the Raman spectra of methane hydrates prepared by ice seed method in the absence/presence of ethanol. Besides, we also synthesized binary ethanol-methane hydrate from stoichiometric 8.6 wt% (5.6 mol%) ethanol aqueous solution at constant pressure 16.65 MPa and constant temperature −15 °C for 24 h with magnetic stirrer at 600 rpm and collected the Raman spectrum. The Raman spectrum of the methane hydrate formed by 8.6 wt% ethanol aqueous solution, as the black spectrum (a) illustrated in Fig. 6, was similar to that of Yasuda *et al*.^43^. The ratio of peak areas of large cavity to small cavity was about 0.44, also consistent with that of Yasuda *et al*.^43^. Indeed, the binary ethanol + methane hydrate prepared by ethanol aqueous solution would form structure II hydrate, consistent with previous studies^8,43,44^.

There is no doubt that the methane hydrate prepared by the ice seed method in the absence of ethanol can only form structure I hydrate, as the red spectrum (c) illustrated in Fig. 6, which is quite different from the spectrum (a) in Fig. 6 for structure II hydrate. The Raman spectrum of methane hydrate prepared by ice seed method in the presence of ethanol vapor, blue spectrum (b) in Fig. 6, was similar to that of methane hydrate in the absence of ethanol, red spectrum (c) in Fig. 6. Even the ratio of peak areas of large cavity to small cavity for spectrum (b) in Fig. 6 was 2.97, in good agreement with that for spectrum (c) 2.83. Note that Lee and Kang^44^ also reported that the sI methane hydrate was observed coexisting with the sII binary ethanol-methane hydrate when the ethanol concentration was as low as 2.0 mol%, way below the stoichiometric concentration 5.6 mol%. The amount of sI methane hydrate would increase along with a decrease in ethanol concentration. That implies that the sI methane hydrate would be formed whenever the amount of ethanol is insufficient to stabilize all the water molecules to form the sII hydrate. Not to mention that the ethanol concentration in the ice powder used in this study was zero to start with and build up via adsorption from the vapor phase. It is believed that the ethanol concentration in the ice powder is too small to stabilize the sII binary ethanol-methane hydrate. As a consequence, the methane hydrate prepared by ice seed method in the presence of ethanol vapor would form structure I hydrate only. In other words, the ethanol vapor can kinetically promote the methane hydrate formation/dissociation rate without changing the hydrate structure and with no ethanol encapsulated in the methane hydrate.

## Conclusion

In this study, ice seed method^27^ was applied to examine the kinetic effect of ethanol on methane hydrate formation and dissociation. Ice was granulated by blender into small particles and sieved to desired particle diameter ranging from 180 to 250 μm. Ice powder (~10 g) was loaded in the sample container and then introduced into the reaction tank and ~2 ml ethanol was added at the bottom of reaction tank with no direct contact between liquid ethanol and ice powder. The reaction tank was pressurized up to 16.65 ± 0.20 MPa by methane. The ethanol liquid would evaporate into vapor phase to form ethanol-doped methane gas. Experimental temperature was ranging from −1.0 to −14.0 °C. The temperature and pressure in the reaction tank were maintained at constant for 24 h to react methane with ice powder to form methane hydrate. After 24 h, the reaction tank was depressurized to atmospheric pressure and connected to flow meter to determine the amount of methane released from the dissociation of methane hydrate. The dissociation process would maintain at the same temperature as the reaction temperature for converting methane hydrate back into ice for 9 h and then the temperature of reaction tank was raised up to 15 °C to dissociate all the ice and methane hydrate remaining inside the system. The hydrate conversion ratio and the rate of dissociation of methane hydrate can be calculated by gas volume released from the dissociation of methane hydrate. It is interesting to find out that the methane hydrate conversion ratio for the reaction time of 24 h is a rather weak function of the reaction temperature ranging from −1.0 to −14.0 °C. The average methane hydrate conversion ratio for the system in the presence of ethanol vapor was 0.801, much larger than that in the absence of ethanol vapor, 0.266. In addition, Raman spectroscopy was used to identify that the methane hydrate prepared by ice seed method in the presence of ethanol vapor would form structure I hydrate only. In other words, the ethanol vapor was a kinetic promoter for methane hydrate formation with no ethanol encapsulated in the methane hydrate. Furthermore, the ethanol vapor could act as a kinetic promoter of the dissociation of methane hydrate by suppressing the self-preservation effect in the dissociation process of methane hydrate system. Almost all the methane hydrates would dissociate in the first hour of the dissociation process in the presence of ethanol doped system.

## Methods

### Apparatus

**Figure 7:**
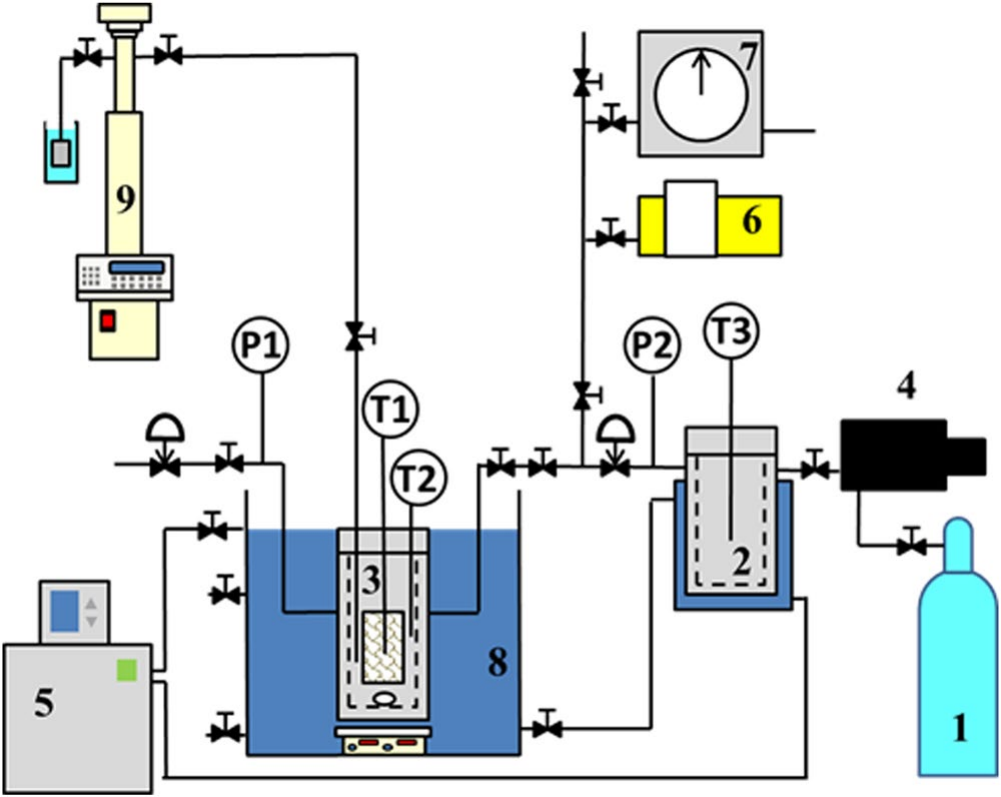
Schematic setup of the experimental apparatus for modified ice seed method. 1, methane gas cylinder; 2, reservoir vessel; 3, reaction vessel; 4, gas booster; 5, refrigerated circulator; 6, vacuum pump; 7, wet gas flow meter; 8, ethylene glycol aqueous solution bath; 9, high pressure syringe pump.

The experimental apparatus is schematically illustrated in Fig. 7. The experimental system consists of a reservoir vessel and a reaction vessel. Two temperature (RTD PT100) probes with an accuracy of 0.1 K, as marked by T1 and T2 in Fig. 7, were equipped into the reaction vessel to measure the temperature of inside and outside the ice/hydrate sample in the reaction vessel. Hereafter the temperatures measured by the probe T1 and T2 are defined as sample temperature and gas temperature, respectively. The pressure was monitored by a pressure transducer (Wika A-10) with a resolution of 0.01 MPa. The system temperature in the reaction and reservoir vessels was maintained and controlled by a refrigerated circulator (Thermo Scientific, PC200-A40). Methane gas was pressurized into the reservoir vessel to about 35.0 MPa by a gas booster. Two pneumatic valves were used to control the pressure in the reaction vessel. One was installed in-between the reservoir vessel and the reaction vessel and the other one was installed right after the downstream of the reaction vessel. When the pressure was lower than 16.35 MPa the pneumatic valve between the reservoir vessel and reaction vessel would open to compensate the methane pressure. When the pressure was higher than 16.75 MPa, the pneumatic valve on the downstream of the reaction vessel would open to release the methane gas. As a consequence, the pressure would be always maintained at 16.55 ± 0.20 MPa.

### Procedure

**Figure 8:**
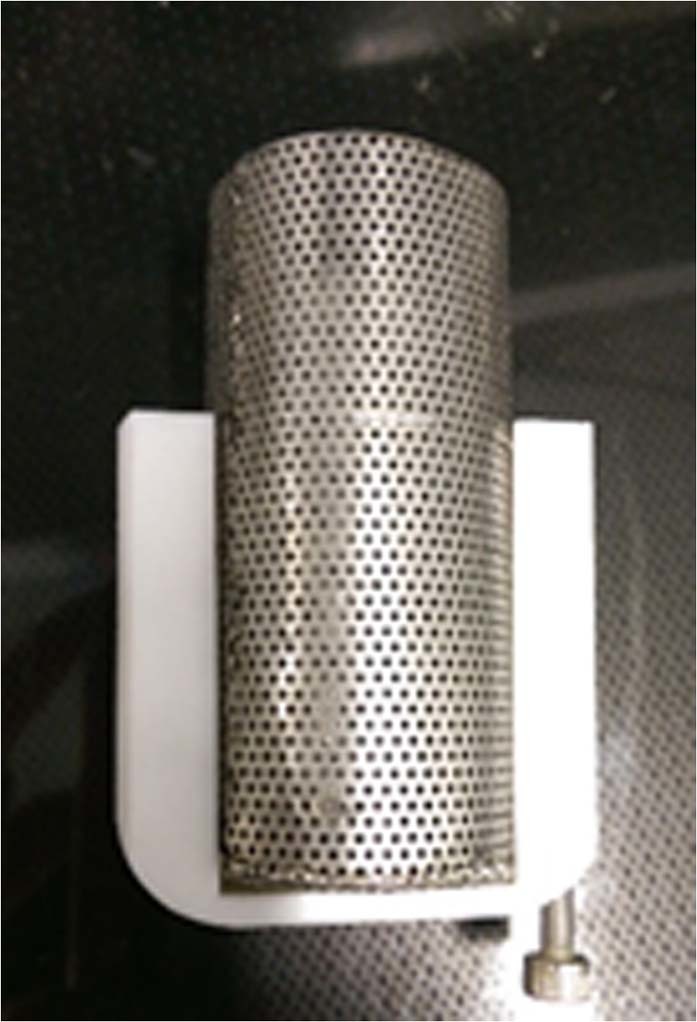
Photo of the ice powder sample container with its holder.

Ice was granulated and sieved to a desired particle size ranging from 180 to 250 micrometers. The ice powder of ~10 g was loosely packed in a stainless steel wire mesh sample container (internal volume of 55 cm^3^), as illustrated in Fig. 8, with a porosity of 75% to ensure substantial amount of solid (ice) surfaces exposed to methane gas for hydrate conversion during the reaction and dissociation stage and then introduced into the reaction vessel. After placing ice powder sample container into the reaction vessel and sealing the reaction vessel, the temperature of the reaction vessel was controlled at a prescribed temperature by the ethylene glycol aqueous solution bath. The air remained inside the reaction vessel was removed by a vacuum pump (ULVAC, GCD-050XA). For some experiments, around 2 ml of ethanol was added at the bottom of the reaction vessel to examine the kinetic effect of ethanol on methane hydrate formation and dissociation. For some experiments, ethanol was injected into the reaction vessel under high pressure using a high pressure syringe pump (Teledyne ISCO, 260D). Note that there was no direct contact between liquid ethanol and ice powder because there was some space between the sample container and the bottom of the reaction vessel. The system pressure was pressurized to 16.65 ± 0.20 MPa by methane and experimental temperature was ranging from −14.0 to −1.0 °C. The temperature and pressure in the reaction vessel were maintained constant for 24 h to transform ice into methane hydrate. After a reaction time of 24 h, the system in the reaction vessel was depressurized to atmospheric pressure and then connected to the wet gas flow meter (Shinagawa, W-NK-0.5, a resolution of 10 ml) to determine the amount of methane released from the dissociation of methane hydrates. The dissociation process would maintain at the same temperature as the one converting ice into methane hydrate but at atmospheric pressure for 9 h and then the system temperature in the reaction vessel was raised up to 15 °C to ensure dissociating all the hydrates remained inside the reaction vessel. We also conducted the experiments with different reaction times (from 1 to 8 h) at a fixed temperature (−2.7 °C) to explore the kinetics of the conversion ratio of ice powder into methane hydrates. In the dissociation process, the temperature would keep at the same temperature (−2.7 °C) for 3 h and then compel all the hydrates to dissociate by raising temperature to 15 °C.

The methane hydrate sample synthesized with and without ethanol added were further analyzed by Raman spectrum. The Raman system includes a Raman spectrometer with a focal length of 1000 mm (FHR 1000, Horiba), Raman probe (Superhead, Horiba) with charge-coupled device (CCD) camera (SYN-1024 × 256-OE, Synapce), and high power laser (532 nm wavelength and 100 mW). The objective was 50 × (M Plan Apo SL50x, Mitutoyo), the slit was 300 μm controlled by the spectrometer. The filter was 532 nm laser line filter provided by omega optical. Each acquisition time was 30 second with two accumulations using an 1800 groove mm^−1^ grating with a spectral resolution of 1 cm^−1^. The Raman spectrum with wavenumber ranging from 2850 to 2950 cm^−1^ was collected.

## References

[1] Sloan, E. D. & Koh, C. A. *Clathrate hydrates of natural gases*. (CRC Press, 2008).

[2] Falenty A, Kuhs WF, Glockzin M, Rehder G (2014). “Self-preservation” of CH_4_ hydrates for gas transport technology: pressure–temperature dependence and ice microstructures. Energy Fuels.

[3] Hammerschmidt EG (1934). Formation of gas hydrates in natural gas transmission lines. Ind. Eng. Chem..

[4] Ng H-J, Robinson DB (1985). Hydrate formation in systems containing methane, ethane, propane, carbon dioxide or hydrogen sulfide in the presence of methanol. Fluid Phase Equilib..

[5] Afzal W, Mohammadi AH, Richon D (2007). Experimental measurements and predictions of dissociation conditions for carbon dioxide and methane hydrates in the presence of triethylene glycol aqueous solutions. J. Chem. Eng. Data.

[6] Mohammadi AH, Afzal W, Richon D (2008). Experimental data and predictions of dissociation conditions for ethane and propane simple hydrates in the presence of distilled water and methane, ethane, propane, and carbon dioxide simple hydrates in the presence of ethanol aqueous solutions. J. Chem. Eng. Data.

[7] Zhurko FV, Manakov AY, Kosyakov VI (2010). Formation of gas hydrates in the systems methane–water–ROH (ROH = ethanol, n-propanol, i-propanol, i-butanol). Chem. Eng. Sci..

[8] Anderson R, Chapoy A, Haghighi H, Tohidi B (2009). Binary ethanol−methane clathrate hydrate formation in the system CH_4_-C_2_H_5_OH-H_2_O: phase equilibria and compositional analyses. J. Phys. Chem. C.

[9] Makiya T (2010). Synthesis and characterization of clathrate hydrates containing carbon dioxide and ethanol. PCCP.

[10] Sloan, D. *et al*. *Natural gas hydrates in flow assurance*. (Gulf Professional Publishing, 2010).

[11] Englezos P (1993). Clathrate Hydrates. Ind. Eng. Chem. Res..

[12] Kumar A, Daraboina N, Kumar R, Linga P (2016). Experimental investigation to elucidate why tetrahydrofuran rapidly promotes methane hydrate formation kinetics: applicable to energy storage. J. Phys. Chem. C.

[13] Veluswamy HP (2016). Rapid methane hydrate formation to develop a cost effective large scale energy storage system. Chem. Eng. J..

[14] Horii S, Ohmura R (2018). Continuous separation of CO_2_ from a H_2_ + CO_2_ gas mixture using clathrate hydrate. Appl. Energy.

[15] Kipyoung K, Youtaek K, Hokeun K (2014). Recent advances in natural gas hydrate carriers for gas transportation. JKOSME.

[16] Prasad PSR, Sai Kiran B (2018). Clathrate Hydrates of Greenhouse Gases in the Presence of Natural Amino Acids: Storage, Transportation and Separation Applications. Sci. Rep..

[17] Hashimoto H, Yamaguchi T, Ozeki H, Muromachi S (2017). Structure-driven CO2 selectivity and gas capacity of ionic clathrate hydrates. Sci. Rep..

[18] Yousif MH (1998). Effect of underinhibition with methanol and ethylene glycol on the hydrate-control process. SPE Prod. Facil..

[19] Abay HK, Svartaas TM (2010). Effect of ultralow concentration of methanol on methane hydrate formation. Energy Fuels.

[20] McLaurin G, Shin K, Alavi S, Ripmeester JA (2014). Antifreezes act as catalysts for methane hydrate formation from ice. Angew. Chem.-Int. Edit..

[21] Bobev S, Tait KT (2004). Methanol - inhibitor or promoter of the formation of gas hydrates from deuterated ice?. Am. Miner..

[22] Amtawong J (2016). Propane clathrate hydrate formation accelerated by methanol. J. Phys. Chem. Lett..

[23] Stern LA, Circone S, Kirby SH, Durham WB (2001). Anomalous preservation of pure methane hydrate at 1 atm. J. Phys. Chem. B.

[24] Chen P-C, Huang W-L, Stern LA (2010). Methane hydrate synthesis from ice: influence of pressurization and ethanol on optimizing formation rates and hydrate yield. Energy Fuels.

[25] Hsieh M-K (2012). Predictive method for the change in equilibrium conditions of gas hydrates with addition of inhibitors and electrolytes. Ind. Eng. Chem. Res..

[26] Hsieh M-K (2012). Explicit pressure dependence of the Langmuir adsorption constant in the van der Waals–Platteeuw model for the equilibrium conditions of clathrate hydrates. Fluid Phase Equilibr..

[27] Stern LA, Kirby SH, Durham WB (1996). Peculiarities of methane clathrate hydrate formation and solid-state deformation, including possible superheating of water ice. Science.

[28] Sloan ED, Fleyfel FA (1991). Molecular mechanism for gas hydrate nucleation from ice. Aiche J..

[29] Shepherd TD, Koc MA, Molinero V (2012). The quasi-liquid layer of ice under conditions of methane clathrate formation. The Journal of Physical Chemistry C.

[30] Zhang Z, Guo G-J (2017). The effects of ice on methane hydrate nucleation: a microcanonical molecular dynamics study. PCCP.

[31] Zelenin YM (2003). Effect of pressure on clathrate formation in a water–ethanol system. J. Struct. Chem..

[32] Chen, Y.-A. *et al*. Measurements of diffusion coefficient of methane in water/brine under high pressure. *Terr*. *Atmos*. *Ocean*. *Sci*. **29** (2018).

[33] Ikeda-Fukazawa T, Kawamura K, Hondoh T (2004). Mechanism of molecular diffusion in ice crystals. Mol. Simul..

[34] Peters B, Zimmermann NER, Beckham GT, Tester JW, Trout BL (2008). Path sampling calculation of methane diffusivity in natural gas hydrates from a water-vacancy assisted mechanism. J. Am. Chem. Soc..

[35] Tokunaga J, Kawai M (1975). Solubilities of methane in methanol-water and ethanol-water solutions. J. Chem. Eng. Jpn..

[36] Takeya S (2001). *In situ* x-ray diffraction measurements of the self-preservation effect of CH_4_ hydrate. J. Phys. Chem. A.

[37] Takeya S (2011). Nondestructive imaging of anomalously preserved methane clathrate hydrate by phase contrast x-ray imaging. J. Phys. Chem. C.

[38] Fujimoto A, Sugahara T (2017). Scanning electron microscopic studies on the methane hydrate decomposition using the freeze-fracture replica method. Bull. Glaciol. Res..

[39] Mimachi H (2014). Natural gas storage and transportation within gas hydrate of smaller particle: size dependence of self-preservation phenomenon of natural gas hydrate. Chem. Eng. Sci..

[40] Nakoryakov VE, Misyura SY (2015). Kinetics of dissociation of hydrate systems with alcohol and electrolyte admixtures. J. Eng. Thermophys..

[41] Anderson GK (2004). Enthalpy of dissociation and hydration number of methane hydrate from the Clapeyron equation. J. Chem. Thermodyn..

[42] Handa YP (1986). Compositions, enthalpies of dissociation, and heat capacities in the range 85 to 270 K for clathrate hydrates of methane, ethane, and propane, and enthalpy of dissociation of isobutane hydrate, as determined by a heat-flow calorimeter. J. Chem. Thermodyn..

[43] Yasuda K, Takeya S, Sakashita M, Yamawaki H, Ohmura R (2009). Binary ethanol−methane clathrate hydrate formation in the system CH_4_-C_2_H_5_OH-H_2_O: confirmation of structure ii hydrate formation. J. Phys. Chem. C.

[44] Lee J-W, Kang S-P (2012). Spectroscopic identification on cage occupancies of binary gas hydrates in the presence of ethanol. J. Phys. Chem. B.

[45] Lin F, Sum AK, Bodnar RJ (2007). Correlation of methane Raman ν_1_ band position with fluid density and interactions at the molecular level. J. Raman Spectrosc..

[46] Sum AK, Burruss RC, Sloan ED (1997). Measurement of clathrate hydrates via Raman spectroscopy. J. Phys. Chem. B.

[47] Zhong J-R (2016). Self-preservation and structural transition of gas hydrates during dissociation below the ice point: an *in situ* study using Raman spectroscopy. Sci. Rep..

